# A194 EARLY LIFE WESTERN-TYPE DIET ACCELERATES THE ONSET OF MURINE IL-10 KO COLITIS

**DOI:** 10.1093/jcag/gwad061.194

**Published:** 2024-02-14

**Authors:** M Ren, A Luchak, C Dang, D Philpott, K Croitoru

**Affiliations:** University of Toronto, Toronto, ON, Canada; University of Toronto, Toronto, ON, Canada; Department of Medicine, University of Toronto, Toronto, ON, Canada; University of Toronto, Toronto, ON, Canada; Department of Medicine, University of Toronto, Toronto, ON, Canada

## Abstract

**Background:**

Early life is a critical time for gut microbiome and immune development, including the establishment of proper host-microbe interactions. While exposures to western world environmental factors are associated with inflammatory bowel disease (IBD) susceptibility, the link between environment in early life and later disease onset is unclear. One of the most important western world environmental factors is diet, which can distinctly alter the gut microbiome. We believe an early life western-type diet (WD) can affect disease progression in a murine IL-10 KO colitis model through dysregulated T-cell responses against the microbiome.

**Aims:**

We aimed to characterize how an early life WD given to IL-10 KO mice would affect colitis development and the gut microbiome.

**Methods:**

IL-10 KO mice were housed in specific pathogen free conditions and fed normal chow (NC) or WD *ad libitum,* either between days 10 to 35 (early WD, eWD) or days 35 to 60 (adolescent WD, aWD). Stool was collected from mice at days 35, 56, and 84 for lipocalin-2 (LCN-2) and 16S rDNA sequencing. Mice were sacrificed at day 84, assessing mesenteric lymph node weight, gene expression, colon damage by histopathology, and colon lamina propria leukocyte (LPL) phenotype and cytokine expression by flow cytometry.

**Results:**

Compared to NC and aWD, the eWD fed IL-10 KO mice displayed increased fecal LCN-2 at days 56 and 84, indicating greater inflammation. At sacrifice, eWD fed mice had larger mesenteric lymph nodes and more significant colon damage by histopathological scoring. Colon LPL preparations showed that eWD fed IL-10 KO mice had higher proportions and absolute numbers of effector and regulatory T-cells. Additionally, higher proportions and absolute numbers of those T-cells were IFNγ^+^, IL-17A^+^, and IL-22^+^. qPCR of distal colon tissue revealed increased gene expression of innate and type 3 proinflammatory cytokines such as *Il1b, Tnfα,* and *Il23,* while showing no change in *Il6* and *Il4*. Taxa positively associated with WD were able to establish a persistent niche in eWD fed mice but not in aWD fed mice. An increase in only one specific taxon was associated with eWD at all timepoints while also most strongly correlating with inflammatory markers.

**Conclusions:**

This data suggests that eWD feeding during gut microbiome and immune development is uniquely capable of increasing long-term susceptibility to intestinal inflammation in IL-10 KO mice. This appears to be associated with persistent colonization of potentially more inflammatory taxa. This work provides insight into the potential role of early life environmental risk factors, i.e. WD, in the later development of IBD.

**Funding Agencies:**

CAG, CIHRUniversity of Toronto, Taconic Biosciences

